# Exploring patients' adherence to antibiotics by understanding their health knowledge and relational communication in encounters with pharmacists and physicians

**DOI:** 10.1016/j.rcsop.2023.100372

**Published:** 2023-11-22

**Authors:** Yngvild Kristine Rochette Bergsholm, Marte Feiring, Colin Charnock, Lene Berge Holm, Tonje Krogstad

**Affiliations:** a Department of Life Sciences and health, Faculty of Health Sciences, Oslo Metropolitan University, Oslo, Norway; b Department of Physiotherapy, Faculty of Health Sciences, Oslo Metropolitan University, Oslo, Norway; c Centre for Connected Care, Oslo University Hospital, Oslo, Norway

**Keywords:** Adherence, Antibiotics, Health knowledge, Relational communication

## Abstract

**Background:**

Antibiotics are drugs essential for the treatment of bacterial infections. Widespread and often improper use of antibiotics are driving the emergence of antimicrobial resistance (AMR) globally. A better understanding of the communicated and understood use of antibiotics as well as improved adherence to treatments are needed to meet this public health threat.

**Objectives:**

The aim of the study is to explore how knowledge of antibiotic use is collected and communicated between patients, physicians, and pharmacists, and how patients seek, understand and use available information on antibiotics in adherence to prescribed treatment.

**Methods:**

Seven focus group interviews were conducted with community pharmacists (three groups, eleven participants), physicians/general practitioners (two groups, thirteen participants), and patients (two groups, eight participants) in Norway. Four focus group interviews were conducted offline and three online. The interview data were analyzed using systematic text condensation in a four-step, descriptive and explorative thematic analysis.

**Results:**

Three main themes were developed about patients' adherence to antibiotics: 1. patients' knowledge about antibiotics and AMR; 2. sources of information about antibiotics/AMR; and 3. relational communication. Patient knowledge about both antibiotics and AMR was somewhat limited, and showed considerable variation. Patients relied on the internet, chat sites, printed information, and face-to-face meetings with health professionals for information. Relational communication between patients, physicians, and pharmacists was found to be important in reducing misunderstandings.

Vulnerability, limited time, and lack of communication were barriers to receiving and understanding information during patient-physician encounters. Increased knowledge about antibiotics and AMR may result in better adherence to prescribed medications.

**Conclusions:**

Patients seek information about antibiotics and AMR in three arenas; digital platforms, printed material and face to face encounters. However, patients often misunderstand important facts relating to this issue. Relational communication between patients, physicians, and pharmacists was important to ensure adherence to treatment regimens. Pharmacists are encouraged to use open-ended questions and build upon the information obtained from the physician to provide patients with tailored advice and ensure proper adherence. Pharmacists' contribution is crucial in optimizing antibiotic use and combating AMR, as they are the final healthcare point of contact before treatment initiation.

## Highlights

• The importance of relational communication in patient counseling with attention to patients' vulnerabilities.
• Actionable recommendations for pharmacists to enhance patient motivation for improved adherence to antibiotic treatment.
• The importance of providing patient tailored information to patient needs ensuring adherence to prescription.
• Identifies challenges in providing adequate healthcare assistance in privacy- in a time-constrained environments.

## 1 Background

The importance of antibiotics in treating infections and preventing the spread of harmful bacteria in communities and clinical settings cannot be understated. However, the ongoing surge of antimicrobial resistance (AMR) poses a global health threat.^1^ As prescribers of antibiotics, physicians play a key role in both reducing and optimizing antibiotic use and hospitals are well advised to monitor use and implement antibiotic stewardship programs. The WHO also recommends that pharmacists be integrated into the “full spectrum of health services”.^1^ Pharmacists play a key role in promoting optimal antibiotic use,^2^,^3^^,^^4^ and their involvement represents an important contribution to antimicrobial stewardship in practice.^5^, ^6^, ^7^, ^8^, ^9^ Nevertheless, the professional collaboration between physicians and pharmacists has room for improvement.^10^ Patients are best taken care of when general practitioners and pharmacists collaborate in providing advice.^11^ Norwegian legislation sets out that pharmacists are obligated to ensure correct use of medication^12^ and that they are accessible to the public.^10^

Norway has low use of antibiotics relative to other European countries.13., 14, 15 However, antibiotics are overused in primary healthcare worldwide, and AMR is not just a local or national concern, but also a global one.^16^ There is still a widespread lack of understanding, awareness, and general knowledge about antibiotics, which affects optimal use.^17^,^18^ The correlation between the excessive use of antibiotics and the occurrence of AMR is well established.^6^ Inadequate knowledge may lead to misuse and improper adherence to treatment regimens, contributing to the development of AMR as additional factors.^6^ Waaseth et al.^19^ concluded that in Norway public knowledge of antibiotics was limited in some areas, especially regarding the causes of AMR development.

Common reasons given by patients for non-adherence are lack of knowledge, factors related to their disease and associated treatment, lack of belief in the benefits and efficacy of prescribed treatment regimens, and a lack of social support.^20^, ^21^, ^22^ Previous studies show inconsistent associations regarding the impact of health literacy on adherence. Some studies indicated a relationship between health literacy and adherence to prescriptions,^23^ while others did not.^24^ Further research is needed to better understand the relationship between health knowledge and adherence to prescribed cures.^25^^,^^26^ Health professionals have been shown to insufficiently meet patients' information needs, leading to patients seeking alternative information sources.^27^,^11^ Patients' understanding of the information given is key to making better decisions regarding their own health.^24^^,^^28^, ^29^, ^30^ A systematic review showed that over one third of patients prescribed antibiotics did not correctly adhere to their prescribed antibiotic cure, and one quarter kept leftover antibiotics for future use.^31^ Communication is important for improving use of antibiotics. Enhanced patient-centered communication is highly correlated with better adherence.^22^^,^^30^^,^^32^^,^^33^ The ability to acquire and make use of information, and generate commitment to a treatment regimen are crucial for maximizing adherence to prescriptions.^33^^,^^34^

Pharmacists are hindered in providing patient-centered care due to lack of time and private spaces for dialogue,^35^^,^^36^ limited access to the patient's medical records, and from their dual roles as health care providers and commercial agents.

The aim of the study is to explore how knowledge about antibiotic use is communicated between patients, physicians, and pharmacists, and how patients seek, understand and use available information on antibiotics and how this ultimately affects their adherence to the prescribed antibiotic(s). The research questions are: (1) what do people who have used antibiotic medication know about this type of medication and about AMR; and (2) how patients access information and how is this health knowledge communicated and perceived in encounters with physicians, and pharmacists? This study explore the structure and mechanisms of patient counseling, providing qualitative insights for ongoing discussions on ways to maximize adherence to prescribed cures.

## 2 Methods

### 2.1 Context

This is a qualitative study of three groups: pharmacists, physicians/general practitioners (GP), and patients who have used antibiotic medication. All participants live in central urban areas in the Norwegian South-Eastern health region. Community pharmacies in Norway are privately owned, primarily by three large pharmacy chains. The majority of community pharmacists hold either a master's or bachelor's degree in pharmacy. Community pharmacists are primarily employed in the three large chains and are integrated into the Norwegian health care system, where their tasks include dispensing prescriptions, providing patient counseling and medical advice, as well as mercantile work.

GPs encounter patients and prescribe medications. The GPs are responsible for the follow-up of patients on their list and refer patients to secondary health care if needed. The GP is self-employed on a fee-for-service basis and is subject to national regulation. The National Insurance Scheme and patients are each charged by the GP for service. Everyone registered with a Norwegian social security number has the right to their own GP.

This study was based on seven focus group interviews performed between October 2020 and January 2021 involving three groups of pharmacists, two groups of GPs, and two groups of patients. Due to the COVID-19 pandemic, three of the focus group interviews were performed using Zoom version 1.6.1.^37^^,^^38^ In 2022,the same material was used to describe the positioning of pharmacists in their interaction with physicians and patients when antibiotics were prescribed and dispensed.^11^ The list and details of the focus group participants have been described previously (Table 1 cited from^11^). All interviews and analyses were conducted in Norwegian, and quotes in the results of this paper have been translated into English by one of the authors.

### 2.2 Sampling strategy

Inclusion criteria were patients above the age of 18 who had used antibiotics at least twice over a period of two years, pharmacists with experience of dispensing antibiotics at a pharmacy, and GPs with experience of prescribing antibiotics.

The eight patients were recruited by open invitation (via acquaintances), advertisements (recruitment poster at a pharmacy with associated QR code), and social media (Facebook). The 13 physicians were recruited through the Antibiotic Center for Primary Medicine, which is a national center of competence established on the initiative of the Norwegian Institute of Public Health. The Center aims to promote rational and restricted use of antibiotics in primary health care, and thus reduce the development of AMR. The 11 pharmacists were recruited through managers of pharmacy chains, social media, and open invitations. The participants were compensated to a sum not exceeding 1000 NOK for travel and committed time.

### 2.3 Data collection

The focus group interview guide was developed collaboratively by the authors to address the following research questions: What patients knew about antibiotics, from where patients gained this knowledge and how patients communicated with physicians and pharmacists about relevant health knowledge relating to antibiotics. The “What” and “How” questions were explicit in the guide, while the “Where” questions evolved during the focus group conversations based on participant responses. The open-ended questions in the focus group interview guide were approximately similar for all groups. A pilot focus group interview (bachelor students in pharmacy) was conducted to check whether the questions in the interview guide were understandable. Two researchers conducted the pilot study, as moderator and assistant moderator. The pilot focus group interview was not included in this analysis.

Written consent for participation was obtained from all participants. The physical (in person) focus group interviews were conducted at Oslo Metropolitan University, Campus Pilestredet, Oslo, Norway. The first author, (pharmacist, PhD research fellow) moderated all focus group interviews together with either a pharmacist (PhD) or biologist (PhD) as assistant moderators. There was no prior relationship between the interviewer and participants. Each focus group interview lasted between 60 and 90 min. After each focus group interview, the assistant moderator produced a written summary. The participants were then given the opportunity to comment on the summary.

During the recruitment process, it was ascertained that all focus group participants were well informed about antibiotics. Malterud^39^ uses the term “information power” to indicate that the more information the sample holds, the less data are needed. Information power is determined by topics such as study objectives, sample specificity, use of established theory, and quality of dialogue. The narrow study objective and well-informed participants ensured good quality of dialogue and resulted in rich material with sufficient information power.

### 2.4 Data analysis

The focus group interviews (in person) were audio recorded using a dictaphone app developed by the University of Oslo (Nettskjema-diktafon mobilapp. v. 3.0). The online focus group interviews were video recorded on Zoom version 1.6.1. After recording, the data were stored in the Service for Sensitive Data (TSD), which is a platform for collecting, storing, analyzing, and sharing sensitive data in compliance with Norwegian privacy regulations. The data were then transcribed verbatim and analyzed using reflexive systematic text condensation, a descriptive and explorative thematic analysis consisting of an iterative four-step process.^40^ The analysis was performed manually using numbered lines and color codes. The coders were three pharmacists (with a master's degree/PhD) and one sociologist (with a PhD), and the analysis was a collaborative effort. One of the authors had mainly qualitative experience, two were experienced in both qualitative and quantitative research methods, and one was a research fellow with a quantitatively-oriented master's degree. The first step of the analysis involved coders reading through the transcript. Based on this, they independently started the text coding process to identify preliminary themes which were then discussed. During the next step, the transcripts were searched in detail to identify meaning units, which were sorted into preliminary themes. Meaning units were subsequently sorted into subthemes. Preliminary themes were adjusted during all of these phases. The condensed narrative was generated from the meaning units organized under each theme and subtheme. In the fourth step, an analytical text was produced based on each theme and subtheme.

The theoretical framework of this study was inspired by philosopher Hans Skjervheim who claims that one can never fully understand another person.^41^^,^^42^ He argues for a triangular relationship between “myself”, “the other”, and “the subject matter” in dialogical communication. This relationship contributes mutual participation and engagement between the actors. In this way, “myself” and “the other” have a subject-subject relationship with a common interest in “the subject matter”. Conversely, when dominance is assumed by “myself” and no care or interest is shown from “the other” a binomial relationship with a common theme for exploration cannot be established.

Additionally, Skuladottir and Halldorsdottir’s, work on “sense of control” in vulnerable patients was employed.^43^ They emphasize the significance of “sense of control” in interactions with assertive health professionals and make use of the analytical terms “demoralization”, “remoralization” and “(dis)empowerment” (all quotes page 895 in^45^). “Demoralization” is used to describe “the subjective experience of being weakened mentally and emotionally regarding one's own level of individual and psychological well-being based on such factors as a sense of purpose and confidence in the future”. “Remoralization” is ascribed to situations “where the subjective experience strengthens the individual's mental and emotional sense of psychological well-being”. These analytical terms are used to prequalify empowerment and disempowerment. "Disempowerment" means "the subjective experience of an interpersonal process whereby a person who has power over another is indifferent to the other and abuses that power"; and the act of empowering is conversely based on the philosophy of seeing the patient as an equal autonomous member of the health care team. Skuladottir and Halldorsdottir^43^ build on Werner et al.'s work^44^ on “sense of control” representing a turning point for patient empowerment.^44^ Applying these combined philosophies and analytical concepts in exploring patients' adherence to antibiotics in encounters with physicians and pharmacists has not been conducted previously.

Throughout the analytical process, the authors collaboratively reflected on and returned to the original transcript to ensure that the analysis was based on the responses of the participants, the research questions and the theoretical framework.

**Fig. 1:**
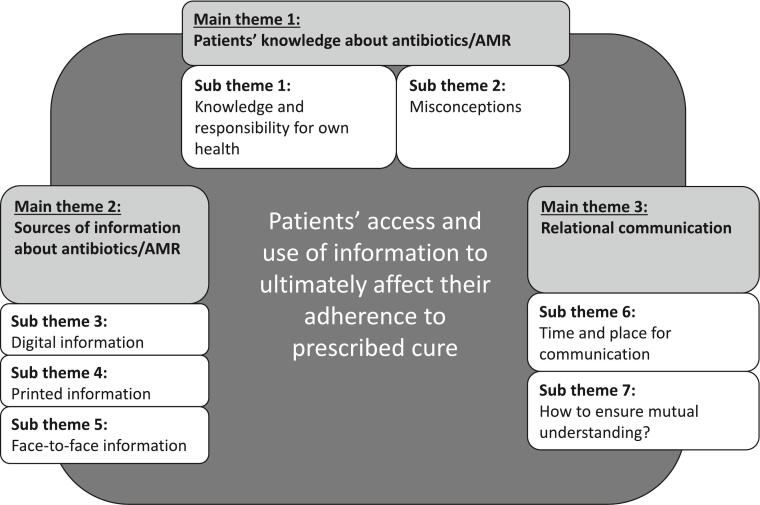
Overview of the main themes and the subthemes emerging from the analysis.

This process developed three main themes and seven subthemes (Fig. 1). The interpretations were discussed collaboratively to increase the trustworthiness of the results. The quotes were translated from Norwegian into English by a native English-speaking biologist PhD who is fluent in Norwegian.

### 2.5 Ethical issues

The Regional Committee for Medical and Health Research Ethics (REK) (ref. number 99284) and the Norwegian Centre for Research Data (NSD) (ref. number 807056) approved the study.

## 3 Results

The analysis developed three main themes: patient knowledge about antibiotics/AMR, their principal sources of information about antibiotics/AMR, and the relational communication in encounters between patients and health professionals (Fig. 1). These themes can be viewed as a basis for patients to improve their knowledge about antibiotics to maximize adherence.

In the results, the code after each quote indicates its origin: a patient (P), a general practitioner (GP), or a community pharmacist (CP). In addition, (m) and (f) indicate male and female, and the number indicates age. A total of 32 participants completed focus group interviews, comprising 11 CPs, 13 GPs, and 8 patients.

### 3.1 Patients' knowledge about antibiotics/AMR

#### 3.1.1 Knowledge and responsibility for own health

The public availability of information about antibiotics allows patients to stay up to date. One female patient explained: “I think that once the doctor has advised and the pharmacist at the pharmacy has advised, then we have the responsibility for taking the medication correctly (…)!” (Pf65). This woman took responsibility for her own health.

In some cases, patients recommended that physicians considered other treatment regimens. One female shared her experience: “After so many years, she understands my problems, and we know that a few days extra sick leave is more effective than a round of treatment with antibiotics” (Pf35). Patients seemed to be satisfied with the available advice on how alternatives to antibiotics could be used to treat infection, e.g., eating cranberries, drinking a lot of water, or trying acupuncture. Many reported benefits from such measures and that they reduced the use of antibiotics, despite not being based on established scientific evidence.

Physicians with a patient centered approach are popular among patients, particularly where patients suffer from repeated infections. Instead of simply repeating the treatment of recurrent infections with antibiotics, they sought more information about the underlying causes, i.e., took a step back and considered the medical history before prescribing antibiotics.

“One way to prevent resistance development is to try to determine the underlying cause of the recurrent infection, and address that, instead of just keeping things in check, which is what I have experienced. It is important to avoid too many rounds of antibiotic treatment” (Pf35).

Many patients had similar experiences and claimed that antibiotics were being prescribed instead of more lasting treatments. They were concerned about being put on repeat treatments without trying find out why infections kept recurring.

#### 3.1.2 Misconceptions

Several patients said they were “afraid of becoming resistant” as a consequence of using antibiotics. They talked about resistance as a disease state or condition that they could acquire from repeated antibiotic use, almost like a side effect: “So in fact, in a little less than a year I've had a few too many kill-or-cure remedies with antibiotics, I've heard I can become resistant to them” (Pf20). The patients regard repeated antibiotic regimens as detrimental. Several patients said that AMR is something that affects the individual and not society. One pharmacist explained: “It seems to me that the belief that people become resistant is quite common” (CPf61). This was confirmed by a physician: “’Is it safe to use antibiotics now, won't I just become resistant?’ Then I have to explain something I find myself doing often” (GPm69). These quotes show that the professionals take responsibility to inform patients about AMR.

Another issue was misunderstandings regarding the dosing regimen. Not all patients understood that “4 times a day” meant approximately 6 h between doses. Patients reported that they combined missed doses. A woman explained her experience:

“I forgot to take it when I was supposed to, so ended up taking two at a short interval. I think if I had been given more information on why this was important, what the reason was, I think I would have taken it more seriously (…). I think what happened was one of the reasons I had to repeat the treatment again later” (Pf20).

Antibiotics are used to treat acute infections as prescribed by a physician. However, accumulation of antibiotic stocks in the home seems to be common. This includes leftover antibiotics when the pack-size does not match the length of treatment, or wait-and-see prescriptions, where antibiotics have been dispensed by the pharmacy, but not used as the patient is advised to wait a given time to see if there is any improvement before taking antibiotics. Having antibiotics available at home made many patients feel safer, and they admitted to being tempted to use them without consulting a doctor. One patient said: “One problem is that you can take your medication home and just have it lying there. Maybe you'll be tempted to use it later” (Pf76). This statement is consistent with one physician's experience: “It's striking that many start using antibiotics before coming to a consultation because they already had some in the cupboard” (GPf54).

A strategy to reduce leftover antibiotics from “wait-and-see” prescriptions is to prescribe antibiotics as “time-restricted medications” with a ten-day validity period. This GP reflects on his practice: “I really think I should have set a time limit for how long the prescription was valid, so it doesn't get used for later infections without the doctor making a diagnosis or giving advice” (GPm65).

Here, the physician saw that he was not consistent. He acknowledged that patients with a new infection could pick up antibiotics at the pharmacy intended for a different infection without consulting a physician, because an older prescription was still valid. This situation was recognized by pharmacists: “The question is, what can you do? It is, after all, still a valid prescription if the doctor hasn't made any restrictions. You can suggest, or get into a dialogue, but you can't deny them the antibiotics” (CPm39).

Many patients did not know that unused antibiotics should be returned to the pharmacy for destruction. Some admitted to having thrown leftover antibiotics in the garbage or toilet. One patient said: “That it's wrong to throw the tablets in the garbage? Oh, I didn't know that, is that because they end up in the environment?” (Pf20).

In summary, patients have a certain level of knowledge about antibiotics and felt responsible to seek more information. They wanted to avoid unnecessary use of antibiotics. Other identified issues were misunderstandings concerning dosing intervals, using leftover antibiotics without consulting the physician, and a lack of knowledge about how to destroy antibiotics.

### 3.2 Sources of information about antibiotics/AMR

#### 3.2.1 Digital information

Where patients obtain their information on antibiotic use varies. Several patients said they used digital information such as HelseNorge (HealthNorway) to supplement information they receive from physicians and pharmacists.^45^ HealthNorway is a Norwegian national online and smart phone application service providing general information about health, disease and prevention of disease, as well as individual information such as active prescriptions and consultations. Patients said they sought digital health information from HealthNorway. A young woman said: “That you can just log on and use HealthNorway if you have any questions about your medication, and it's really good information if there is anything you want to know” (Pf20). An older woman agreed: “Yes, I am one of those who use HealthNorway all the time” (Pf65).

This is in line with what physicians expressed about their patients' use of HealthNorway: “You know, they are logged on HealthNorway and check their prescriptions. Even many of the older patients manage it. They are more data literate than I imagined” (GPf60). However, pharmacists seemed to have a somewhat different experience. Several pharmacists mentioned that patients have problems using HealthNorway as well as understanding and assessing information. One pharmacist explained: “They feel it is difficult to log into HealthNorway and so on (…) And then they just ask us” (CPm45). Several pharmacists also expressed that patients lack the necessary skills to critically evaluate health information sources.

Some patients called for a chat function with a real pharmacist, not a robot, so that they could raise problems relating to antibiotic treatment. One young woman said: “HealthNorway is a good solution, but I think it would be better with an improved pharmacy-based chat service. We end up asking the doctor a lot of unnecessary questions that could be answered online by a pharmacist” (Pf20). The younger patients called for a robust website where they can search for information themselves. They were comfortable with searching for information digitally. However, patients seeking digital information could find it difficult to filter out irrelevant information and felt safer chatting electronically with a pharmacist:

“A chat function, that gives you a real person to ask instead of having to find out everything yourself, (…). The internet can be negative and dangerous! I use digital tools myself, but when it comes to diseases and the like I can't manage on my own because I don't have the necessary training, that's my subjective opinion in any case” (Pf65).

#### 3.2.2 Printed information

Both physicians and pharmacists claimed it was useful to hand out written material to patients. Physicians said that it was beneficial when they knew the patient's medical history: “We are at an advantage because we know almost all our patients. We know who would benefit from getting a letter” (GPf60). In most cases a printout of the medical record or a handwritten note was provided to patients, especially if they suspected cognitive impairment.

Several patients expressed gratitude for printed material and were grateful when the written information was easy to understand: “Because many of us don't understand these fancy words (...) I usually pick up and read the pamphlets, and this gives me the information I need in an easily understandable form” (Pf65). They found it useful to receive pamphlets from pharmacists covering topics such as treatment of recurrent infections, AMR, and information about common types of antibiotics. Patients said that such pamphlets could prepare them for their meeting with the physician:

“We could pick up a pamphlet and put it in our bag. If the pharmacist sees that we have had 10 rounds of treatment over a certain time, then the pharmacist can just say: look here, this shows that you have used this particular medication a lot, have you talked to your doctor about it? It gives the patient something to point to in the next meeting with the doctor” (Pf35).

Another patient expressed their gratitude for pharmacists who offer additional services to patients, such as providing pamphlets explaining the reasons for recurrent infections. “Gosh, they care more than I thought (nodding and smiling)” (Pf65).

The pharmacists discussed language and cultural challenges posed by patients from other ethnic backgrounds. Pictograms were used in counseling and one pharmacist commented as follows:

“It can help those who don't know the language but also those who do know the language but are unsure whether you said 1 tablet 3 times a day. What does that mean, they ask? What is ‘lunch’? Some don't know what ‘morning’ and ‘evening’ mean. Things can also be misunderstood” (CPf25).

#### 3.2.3 Face-to-face information

Regarding the course of their disease, patients were aware of the responsibilities of health professionals and either consulted the physician or pharmacist depending on the information they were seeking:

“It has a bit to do with what kind of questions you have. If it's a simple question where you need a quick answer about the medication, then the pharmacist fits the bill. When you need a deeper answer related to your own person, you are more likely to ask the doctor. It doesn't really matter if the pharmacist provides extra information which I felt was lacking (smiling)” (Pf20).

Information from pharmacists and physicians was advantageous in different areas. Although, patients were just as likely to ask pharmacists questions about antibiotic treatment as the physicians, they preferred discussing sensitive issues with the physician.

Patients mentioned receiving useful information from both the physician and the pharmacist. Both provided practical information about dosage, treatment duration, whether it should be taken with or without food, and other precautions. One patient recounted his previous visit to the doctor: “And he just said that I should start straight away, and that I should take one tablet morning and evening (...) The treatment should last a week, and if I developed a fever or the like I should call him straight away” (Pf31).

During consultations, patients appreciated being informed about their disease and treatment: “It's just that there is too little information [on the label]. I ask and dig if I think something is unclear” (Pf42). Some patients said they prepared sticky notes in advance, which helped them to remember their medical history and the questions they wanted to ask their physician. One patient explained: “I usually note some things down before I go to the doctor in order to remember the questions I want to ask” (Pm77). During consultations, the physician's main task is to pinpoint patient concerns about treatment and symptoms.

In summary, patients want to stay up to date and search for information online, through chat functions, or by reading written information. Coming well-prepared to a consultation is beneficial for an optimal result. Patients used the pharmacist as a source of information but preferred the physician for certain issues.

### 3.3 Relational communication

#### 3.3.1 Time and place for communicated information

Many patients expressed the need for more time in their encounters with health professionals. They welcomed being listened to and having the opportunity to influence their treatment. They emphasized that the consultation is too short: “I have an assigned physician who makes me feel like a burden every time I'm there (…). They don't have the time for that important conversation (…) it's important to discuss things and not just be shown the door with the message to figure it out yourself” (Pf31).

Some patients said they felt pressured to be quick and efficient, which did not provide a good basis for patient-centered communication. When patients felt that healthcare personnel showed an interest in them and treated them with respect, took time to listen and took them seriously, they felt taken care of. This manifested in the use of terms such as “my pharmacist” or “my physician”: “So then I'm happy with my pharmacist” (Pf65). A personal relationship with healthcare personnel establishes trust.

Several methods were used to map patient knowledge about prescribed antibiotics. Physicians and pharmacists did not have time to confirm patients' questions, or allow them to patiently repeat their understanding of the given information which was considered time-consuming in their busy working days:

“Asking patients to confirm and repeat the questions, I have to admit that sheer time constraints are constantly making me move things down the list. I wish it was otherwise, but I find myself thinking about the next customer instead of double-checking if the person in front of me has understood” (CPm42).

This was received support from another physician: “Yes well, I know there is some research suggesting we should ask people to repeat what they said, but time doesn't stretch that far in a busy day, so that is something I don't consider much” (GPf50). This was in line with a patient's experience: “I don't think that has ever happened [about being asked to repeat]” (Pf76).

At the pharmacy, discretion was another issue. One patient urged pharmacists to pay more attention to protecting privacy and to offer, to a greater extent than was current practice, conversations in a separate room: “Patients are sensitive about their diseases, so many don't want those questions in an open space. The pharmacist or the pharmacy technician should then show them the courtesy of taking the customer into a private room to discuss the matter (…)” (Pf65).

#### 3.3.2 How to ensure mutual understanding?

Patients would like healthcare personnel to investigate how much they know about a topic before providing information. Patients are different and have different needs. One young patient said:

“Here you can see four people who all have different wishes concerning what they want from the pharmacist: one has a long history and doesn't need much information, some only want the most necessary instructions, and others want to ask questions. As a person working with people, it is natural to see who should be given what kind of information (…)” (Pf20).

Some patients said they are not interested in being informed about all the medication side effects. One pharmacist said: “They make it clear if they are not interested, and then we have to decide: should we back off, or do we have something so essential to say that we have to try and get the point across” (CPm42). Here, the pharmacist respects the patient's autonomy and balances their obligation to provide counseling under the applicable legislation.

For the pharmacist, it may be unclear what information the physician has given about the prescribed antibiotics. To establish this, patients suggested that the pharmacist should ask: “What kind of information has the doctor given you about this antibiotic?” Some pharmacists have positive experiences of using such an approach to filter out exactly what kind of information the patient needs:

“My approach is to always ask the same question, which is: What kind of information did you get about this medication? This is a suitable question to obtain the (…) patient's knowledge. Then they will normally start telling you. Repeating what the physician has said. And then I have the opportunity to confirm, supplement, or explain” (CPm42).

Such questions were welcomed by the patients: “To me this sounds like a great pharmacist who actually asks pertinent questions about what doctor has said. Questions I would like to be asked” (Pf35). This method ensured that the information was tailored to the individual's needs. In addition, patients were encouraged to be attentive to the information provided by the physician and to verbally phrase this themselves.

“At the same, this encourages better learning. If you ask: what did the doctor say, then the patient has to dig into their memory and this heightens their awareness if they find they haven't actually got any information – this makes them more receptive to the information that we subsequently give them and they are more likely to remember it – A good strategy, I think” (CPm39).

Instead, many patients reported that pharmacists started the patient interaction with a closed question: Have you used this before? If the patient answered yes, then no more information was given, and if the answer was no, the information was then often reeled off on autopilot. During their consultation with the physician, patients felt submissive, and many said they were less receptive to the information that was given. The infection compromised their state of health and made them feel vulnerable. Their primary focus at the physician's office was on being believed and making their symptoms understood. These circumstances made it difficult for patients to process the information given. After the consultation, when patients felt they had been heard and prescribed an antibiotic treatment they were empowered with a hope of achieving a rapid recovery. The patients were more motivated to receive information at the pharmacy.

“I have an assigned doctor who says: now we're going to make you well. Then I feel (using arm movements as she talks) much better when I leave the office than I did when I arrived (mild laughter). I take that positive feeling with me when I arrive at the pharmacy and am more receptive for other types of information that the pharmacist might provide. These are things I've thought a bit about” (Pf35).

Many patients showed an interest in their own illness and recovery, saying it was easier to do as they were told by health personnel when they knew why:

"All these precautions! They should explain what the issue is and the reason for it. It's often easier if you know. You can't do this because… is better than being told not to do so at all (demonstrates with a pointing finger). I think this pedagogic approach, and working on communication, is something pharmacists should consider in their encounters with patients" (Pf35).

One pharmacist supported this view: “And then you're often positively surprised because people are inquisitive; so just that, to get a bit more of an insight into the medical background, gives you more motivation to take your medication” CPm39. The pharmacist explained that when patients are informed and have understood how to do as prescribed, they were more motivated to adhere to the prescription.

One patient continued:

“So it's clear that for very proactive patients, but also for those who are not, then the role of the pharmacist can be strengthened by using a forward-looking mode of communication: Yes, I see that the doctor has said that you should take these four times daily, that's because… do you know that? This is both an informative and pedagogic approach that I support” (Pf35).

Many physicians expressed that the involvement of pharmacists would confuse and disrupt the agreement that they had already made with the patient. One physician said:

“But I think that the pharmacist should also be a little careful to avoid creating unnecessary distress, I've experienced that some people have been given additional information that has either made them afraid to take the full dose or complete the treatment (…)” (GPm42).

Some physicians did not accept that pharmacists' patient-centered communication added value for the patient. One physician supported this view: “Sometimes the patient has called and said that the pharmacy said this and that (agreement in the background), can you phone the patient and explain. In other words, there is a lot of disturbance when information is coming from all directions” (GPf50). The physicians wanted the pharmacists to stick to their traditional pharmaceutical tasks – to ensure that the patient got the right dosage of the right antibiotics at the right time. However, this conflicted with the patients' wishes. An older patient explains: “Any and all repetition provided by the pharmacist would only be an advantage” (Pm77).

In summary, patients expressed that they felt vulnerable and that physicians lacked the time to give them information. After consultation with the physician, patients expressed motivation for receiving information about starting antibiotic treatment. The receptiveness of patients to information at the pharmacy, and their need for knowledge provides an opportunity for the pharmacist to take action. However, physicians are concerned that the information provided by pharmacists may be in conflict with the information they provide, and that this might be confusing for patients.^11^

## 4 Discussion

The three main themes illustrated in Fig. 1, Patient's knowledge about antibiotics/AMR, sources of information about antibiotics/AMR, and relational communication, are factors that influence patients' adherence to the prescribed antibiotic cure. Although patients have some knowledge about antibiotics and feel responsible for own health, misconceptions about the use of antibiotics and AMR development exist. There is a lack of patient-centered information at both physician's offices and pharmacies.

### 4.1 Knowledge about antibiotics/AMR

Knowledge in this context is related to what Skjervheim refers to as the “subject matter”.^41^^,^^42^ When health professionals inform about optimal antibiotic use, adherence to the medication, and AMR, this should be done in a way that engages the patient. If the patient participates together with the physician and pharmacist and a triangular relationship is formed^41^^,^^46^^,^^47^ a mutual participation and engagement develops between the actors. This collaborative approach can lead to enhanced knowledge, shared understanding and increased adherence to the treatment.

In this study, patients have some information about antibiotics and AMR. However, their knowledge is limited in some areas, a finding supported by other studies.^19^^,^^48^^,^^49^ One reason for misuses and lack of adherence to prescribed medications, were the patients' lack of health knowledge, which is a product of gender, age, level of education, and long-term illness.^50^ For example, Le et al.^50^ showed that digital information is easily accessible, but emphasized the importance of knowledge to understand the information. Patients with little formal health knowledge reported that they experienced poor treatment in the healthcare system. According to Hristov and colleagues, there is a link between low health literacy and patients' ability to express themselves about their health problem. This in turn can result in poorer health.^51^

People's level of health knowledge and their ability to acquire and understand information differ, which could lead to misconceptions. Williams^52^ reported a discrepancy between what patients understand and what professionals think they understand. Standard information to patients is often given at a higher level of health literacy than many patients typically possess.^52^ This is in line with our results, where patients claimed that pharmacists and physicians sometimes “talked over their heads”. This study emphasizes the need for healthcare professionals to create an environment where patients are heard, respected, and valued as partners in their own care. Then, in Skjervheim's framework for dialogical communication,^42^a triangular relationship between physician or pharmacist / "ourselves", patient / "the other", and medical concern/“subject matter" can be achieved and lead to joint exploration of the patients health literacy to improve its quality to maximize adherence to prescribed cure.

Another reason for misconceptions, seen both in the present study and in that of Williams,^52^ was that healthcare professionals did not fully comprehend the challenges patients faced when it came to understanding the available information. Again, in Skjervheim's framework,^41^^,^^42^ when healthcare professionals dominate the dialogue and are not interested in what the patient has to say, the relationship becomes asymmetric, without common theme for exploration.,

Availability of online health information to patients has been shown to increase patient empowerment.^53^^,^^54^ When patients are empowered with health information regarding their clinical situation, they can become “experts” on their own disease,^55^ which empowers them in their encounters with professionals.^56^^,^^57^ Being empowered helped patients to collaborate with healthcare professionals in a medical decision-making process,^58^^,^^59^ and facilitated Skjervheim's^41^^,^^42^ triangular relationship, where health professionals and patients are encouraged to engage in constructive dialogue on the “subject matter”. Broom^59^ writes that online information has a potential to empower patients, which resulted in greater control of their disease. These results are supported in this study. Patients were prepared in advance, utilizing various tools including digital personal and general health information from Health Norway.

Bowes and colleagues^60^ found that patients tended to give priority to the physician's opinion over the information obtained from online information, if the physician showed interest and spent time on the patient's medical history. In our study, patients said that they prepared in advance by using online information to empower themselves for their encounters with professionals. They also made use of own written notes to help them remember medical history and relevant questions. Patients asked questions in a prioritized order, starting with those most pertinent to their disease. Being well-prepared for encounters with professionals was associated with optimal results. When both the healthcare professional and the patient were engaged in a dialogue where they actively listened to each other and explored a common theme, it led to a more collaborative and participatory relationship which in turn can secure adherence to medications.

### 4.2 Relational communication and a sense of control

In the material presented here, many patients expressed a sense of vulnerability during their consultation with the physician. Some patients experienced a loss of control and disempowerment. Skuladottir and Halldorsdottir^43^ have highlighted how women with chronic pain can be both empowered or disabled by health professionals. In their study, healthcare professionals were seen as potentially powerful actors. Because of the imbalance in power and their physical symptoms patients experienced a sense of being emotionally and mentally impaired when it came to their individual and psychological well-being.^43^ During consultation with the physician, patients in the study conducted by Skuladottir and Halldorsdottir^43^ reported that their focus was mainly on the possibility for recovery and securing the physician's understanding and opinion. The patients in our study were acutely ill with an infection that could have increased their sense of vulnerability and stress. They said that in this setting, they were less receptive to embracing new information. Also, Nafradi et al.^61^found that when patients were listened to, and their symptoms were taken seriously, they were empowered and motivated to adhere to their prescriptions.^61^

Relational communication, based on a triangular relationship, does not harmonize with the use of pre-made checklists, which contribute to standardized information procedures^62^^,^^63^ and does not necessarily meet the patient's information needs at the pharmacy.^36^^,^^64^^,^^65^ This aligns with the perspectives of patients in this study, where information was perceived as being presented in an “autopilot manner”. The patients had different diseases and varying information needs. Some patients wanted their antibiotics as quickly as possible, while others sought more detailed information about the prescribed medication. The pharmacists had to strike a balance between respecting patient autonomy and maintaining information requirements.^10^^,^^12^ The fact that patients were receptive to information from the pharmacist, with knowledge about different medical treatments, provided an opportunity for the healthcare system to take action and involve the pharmacist.^1^ This is considered is important for identifying antibiotic-related problems and ensuring adherence to the prescription.^66^, ^67^, ^68^

Pharmacists face the challenge of balancing their dual roles as healthcare professionals and commercial actors. Limited time for patient-centered counseling and privacy in pharmacies often hinders the pharmacist's ability to offer comprehensive advice.^69^^,^^70^ The patients viewed pharmacists as an untapped resource that can help them to understand why adherence to the prescription is important and thus contribute to an optimized use of antibiotics. These results are supported by several previous studies.^68^^,^^71^, ^72^, ^73^, ^74^ Relational communication skills among pharmacists have an impact on health outcomes, patient satisfaction and adherence.^75^ Here, patients communicated that they felt empowered when pharmacists and physicians collaborated in their medical counseling.

### 4.3 Strengths and limitations

Trustworthiness in qualitative research includes evaluations of quality, rigor, and relevance.^76^ Three of the contributing authors are skilled and trained qualitative researcher experienced with methodological and analytical approches. Two authors participated in the focus group interviews, one interviewer and one observer. The authours have different educational backgrounds, providing different perspectives on the method process and conceptual framework. The flexible use of a focus group interview incorporated explicit “What” and “How” questions allowed “Where” questions to emerge and be elaborated from participant responses. Together the applied approch enhanced the trustworthiness of our study.

In the focus group interviews with pharmacists and physicians some participants were previously acquainted, which would be expected to influence group dynamics. By allocating time to establish a safe environment in the beginning of the interview, these effects were mitigated. There were more females than males in the patient groups as is often the case in similar qualitative studies..^77^, ^78^, ^79^ Recruitment to focus groups based on volunteering may bias participation towards those with a particular interest in the topic. All the physicians and pharmacists were recruited from central urban areas of south-eastern Norway. Such a selection may mean rural settings are underrepresented. However, whether the location (central or rural) could influences pharmacy counseling has been discussed in the literature, and generally, it has been concluded that location is not necessarily a primary factor.^80^, ^81^, ^82^

Homogeneous focus groups of patients, physicians, and pharmacists were chosen to avoid potential skeweddistribution of power within each group. The asymmetry in the hierarchy ranking could hamper the discussion within a heterogeneous group.^38^^,^^83^ Interactions within the group are crucial for obtaining a rich qualitative material..^4^ The decision was, therefore, made to also recruit pharmacists and physicians who knew each other prior to the focus group interviews. In the first focus group with pharmacists, a pharmacy manager and two of her employees took part. This composition affected the dynamics of the group. The employees looked at their manager as they spoke, letting her speak before them. When the interviewer was made aware of this, the effect was reduced by actively giving the floor to an employee before the manager. This may nonetheless have affected how honest the employees were in their answers. The study compensated participants for their time and travel expenses. Financial compensation for study participation may bias the recruitment,^84^ but was here considered minor and fully outweighed by the necessity of obtaining relevant informants.^85^

### 4.4 Implications

This study explores patients', physicians' and pharmacists', knowledge and experiences of antibiotic use and AMR. Seen from the viewpoint of the patients, a greater understanding of the propriety of antibiotic adherence to treatment is considered necessary for following recommendations for improved adherence, and thus make a contribution to the broader effort of combating AMR.

The study also explores how pharmacists struggle to employ a patient-centered approach and build upon the information obtained from physicians to provide patients with tailored information for maximizing adherence. Additional research is recommended to investigate the feasibility of implementing these suggestions and their potential for maximizing adherence. The first author is currently implementing and evaluating a tailored practical information to pharmacists based on the results of this and other studies.

Another result addresses the challenge of providing adequate healthcare assistance and maintaining the need for patient privacy in a time-constrained environment, all the while balancing the financial needs of the business. More research on the pharmacist's dual roles is recommended.

Physicians in this study discussed the challenge of prescribing antibiotics with either time-restricted validity (10 days) or as needed medication (12 months validity). In the future, the usefulness of this practice needs to be investigated.

## 5 Conclusion

This study shows that patients enact varying levels of health knowledge about antibiotics and express a sense of responsibility to stay informed. They actively seek information about antibiotic use from three main sources: digital platforms (online, including chat forums), printed materials, and face-to-face interactions with health professionals. Despite this, misunderstandings and improper use of antibiotics continue to happen. By applying analytical concepts from the frameworks of Skjervheim and Skuladottir and Halldorsdottir, this study indicates that relational communication is important in patient-centered care in vulnerable situations.

## Funding

The Foundation for the Promotion of Norwegian Pharmacy funded this study.

## Author contributions

YKRB, LBH, CC, MF and TK substantial contributed to the design and conception of the work. YBRB, LBH, CC and TK were involved in the data collection. YKRB, MF, LBH and TK performed the analysis and were involved in the interpretation of the data. YKRB drafted the article and LBH, MF, CC and TK critically revised the article. The final version of the manuscript was approved by all the authors, and all authors agree to be accountable for all aspects of the work.

## Declaration of Competing Interest

No conflicts of interest exist. The authors alone are responsible for the content and writing of this article.

## Appendix A Supplementary data

Supplementary material Image 1
