# Supplementary material for: Exploring patients' adherence to antibiotics by understanding their health knowledge and relational communication in encounters with pharmacists and physicians
Source: Explor Res Clin Soc Pharm. 2023 Nov 22;12:100372. doi: 10.1016/j.rcsop.2023.100372 (PMC10711180; doi:10.1016/j.rcsop.2023.100372)
Supplement: Supplementary file 1 — Supplementary material [file mmc1.docx]

Interview guide: Information flow between General Practitioners (GP), the patient and Community Pharmacist (CP) concerning antibiotic use, handling and resistance.

1. What information is given about the use and handling of antibiotics?

o To the GPs: When antibiotics are prescribed to a patient: What information is given about the use and handling of the medicine?

o To the CPs: What information do you think doctors usually give to patients when prescribing antibiotics? (What kind of counselling do you give them)?

o To the patients: What information do you get from the doctor when you get a prescription for antibiotics?

2. Information on antibiotic resistance

o To the GPs: What information do you give about antibiotic resistance to the patient when you prescribe antibiotics?

o To the CPs:

What information do you think the doctor has given about resistance to patients who have been prescribed antibiotics?

What information do you give about resistance to the patient when you dispense antibiotics?

o To the patients: What information do you get about resistance when you get a prescription for antibiotics?

3. How do you make sure that the patient has received the essential information?

o To the GPs: How do you make sure that the patient has understood the information you have given?

o To the CPs:

How do you converse with the patient about what information the patient has received from the doctor?

How do you ensure that the patient has understood the information given when dispensing antibiotics?

o To the patients:

How does the doctor ensure that you have understood the information he/she has given you?

How does the pharmacist ensure that you have understood the information he/she has given you?

4. What information do you think patients really need and want?

To the GPs: What information do you think patients really need and want?

To the CPs: What information do you think patients really need and want?

To the patients: What information do you need and want to receive from your doctor and pharmacist when you receive antibiotics

5. Wait And See Prescription:

How do you think this arrangement works?

What kind of control questions are asked?

What kind of information is given about antibiotic resistance?

How many patients do you think immediately collect the prescribed antibiotics?

6. Written information:

What kind of printed information is usually provided?
